# Circular Medical Association

**Published:** 1853-08

**Authors:** 


CIRCULAR MEDICAL ASSOCIATION.

The Treasurer of the American Medical Association makes the following statements, which we hope will arrest the attention of all our readers. More subscribers are wanted to the Transactions of the Association. We solicit the co-operation of every physician who desires the advancement of his profession. These Transactions are of great interest and ought to be in every medical library in our country

PHILADELPHIA, *June* 14, 1853.

*To the Medical Profession:*—In conformity with a resolution of the American Medical Association it becomes my duty to inform you of the decision of the Committee of Publication in regard to the price at which the forthcoming volume of Transactions will be furnished to the members of the Association and to others.

The price of *single copies* has been fixed by the Association at *five dollars.* Any *individual* desiring two copies, will be furnished with them upon his remitting to the Treasurer *nine dollars.*

Societies, Associations, and Institutions requiring *six* copies for the use of their members, will be supplied with them on remitting to the Treasurer *twenty-five dollars,* or they will be supplied with twenty-five copies on remitting *seventy-five dollars.*

Those who wish volumes 5 and 6, may obtain them by remitting *eight dollars.*

In consequence of the numerous illustrations, many of them highly finshed colored lithographs, with which the forthcoming volume will be accompanied, its cost will considerably exceed that of either of those previously issued by the Association. To defray the cost of its publication, at least *fifteen hundred dollars,* in addition to the amount already received by the Treasurer, will be required

Your attention is called to the following resolution adopted at the last session of the Association.

"*Resolved,* That the Delegates from the several States be requested to appoint committees, who shall aid the Committee of Publication in procuring subscribers for, and in distributing the Annual Transactions of the Association." Respectfully, D. FRANCIS CONDIE,

***Treasurer and Chairman Com. of Publication A. M. A.***

